# Editorial: Natural products as potential therapeutics to tackle life-threatening infections: From field to market

**DOI:** 10.3389/fphar.2022.1099181

**Published:** 2022-12-07

**Authors:** M. Mukhlesur Rahman

**Affiliations:** The Medicines Research Group, School of Health, Sports and Bioscience, University of East London, Stratford Campus, London, United Kingdom

**Keywords:** antimicrobial resistance, natural products, antimicrobial compounds, antibacterial compounds, antifungal compounds, antiviral compounds, nanoparticles

The Research Topic was intended to focus on the antimicrobial natural products that have revealed significant potentials to life-threatening infections. Considering the catastrophic consequences of antimicrobial resistance (AMR) in today’s healthcare system around the globe, it is no doubt important to look into naturals products (e.g., medicinal plants, microoranisms and animals) for the discovery of new antibiotics with therapeutic efficacies against multi-drug resistance bacteria (Rahman and Sarker, 2020). Accordingly, this Research Topic has invited articles in the recent discoveries of antimicrobial therapeutic agents including bioassay directed isolation and identification of anti-infective compounds from natural sources (medicinal plants, microbes, marine organisms and animal), their mode of actions as potential anti-infective agents, anti-infective drug development from natural resources as well as anti-infective clinical studies on herbal medicines. The overall aim of this research topic was to gather the scientific data on the recent advances on antimicrobial drug discovery from natural sources.

This Research Topic covers a wide range of articles (8 in total) including four original articles, two review articles, one systematic review and one method. Four original articles for this Research Topic have focused on the secondary metabolites from natural resources. Hu et al. explore a traditional Chinese medicine (TCM) preparation, Kumu, for bioassay directed characterization of antimicrobial compounds using an UHPLC-Orbitrap-Ion Trap Mass Spectrometer, combined with MS Fragmenter and ChromGenius. Kumu contains predominantly the stem of *Picrasma quassioides* (D. Don) Benn (Fam. Simaroubaceae). Being effective against microbial infection, inflammation, fever and dysentery, Kumu is widely used in China in the form of tablet, capsule and injection for the treatment of influenza, upper respiratory tract infections, acute tonsillitis, enteritis, and dysentery. Using bioassay directed method, authors have reported 25 active compounds (24 alkaloids and one triterpenoid) from the methanol extract with activities against *S*. *aureus* but only two compounds (β-carboline-1-carboxylic acid and picrasidine) being active against *E*. *coli*. Moreover, three β-carbolines (methylnigakinone, nigakinone and β-carboline-1- carboxylic acid) have also been assessed for their IC_50_ and MBC. Among these three compounds, methylnigakinone’s antimicrobial activity was reported in a Chinese patent against *Staphylococcus aureus*, *E. coli*, *Pseudomonas aeruginosa*, *Streptococcus hemolyticus* and *Streptococcus pneumoniae*. This study has utilized UHPLC-Orbitrap-Ion Trap Mass Spectrometer combined with MS Fragmenter and ChromGenius for the analysis of compounds. The antimicrobial components reported in this study have reasonably justified the medicinal usage of Kumu in TCM. The analytical techniques used in this study has also provided the basis for the analysis of complex chemical compounds in herbal medicine and thereby, contribute to the scientific development of quality standards for TCM.

Zhang et al. describe both *in-vitro* and *in-vivo* anti-HBV activity of a polysaccharide isolated from *Eupolyphaga sinensis* Walker (Fam. Corydiidae) which has been reported to enhance immune response and promote blood circulation by removing blood stasis and therefore, is incorporated in clinical natural healthcare products in China. In this study, the authors have used hepatoma cell line HepG2.2.15 and C57BL/6J HBV-transgene mice as experimental models *in vitro* and *in vivo*. The key finding of this study is significant inhibition of HBV antigen, HBV DNA, and HBV core protein formation through TLR4 pathway by *Eupolyphaga sinensis* polysaccharide (ESPS) with adequate scientific evidence of ESPS as an anti-HBV agent. Simpson et al. report the therapeutic efficacy of soluble non-starch polysaccharides (NSP) derived from plantain (*Musa* x *paradisiaca* L., Fam Musaceae) on the *Clostridioides difficile* infection (CDI)- a leading cause of antibiotic-associated diarrhoea. Considering the fact that dietary fibres are beneficial for intestinal health, the authors previously reported the ability of soluble non-starch polysaccharides (NSP) from plantain banana to block epithelial adhesion and invasion of a number of gut pathogens (e.g., *E*. *coli* and *Salmonellae*). In this article, the authors have evaluated the action of plantain NSP and a range of alternative soluble plant fibres for inhibitory action on epithelial interactions of *C*. *difficile* clinical isolates, purified endospore preparations and toxins. The study has revealed the ability of plantain NSP from plantain, broccoli and leek to disrupt epithelial adhesion of *C*. *difficile* vegetative cells and spores. However, they found NSP of plantain are more potent than those of broccoli and leek. The plantain NSP has significantly diminished the epithelial impact of *C*. *difficile*, reducing both bacteria and toxin induced inflammation, activation of caspase 3/7 and cytotoxicity in human intestinal cell-line and murine intestinal organoid cultures which suggest that soluble plantain NSP may provide a protective effect in CDI patients. Such plantain soluble dietary fibre may therefore be considered as a therapeutically effective nutritional product to treat or prevent CDI and antibiotic-associated diarrhoea.

Dudau et al. report a fatty acid fraction separated from sea buckthorn (*Hippophae rhamnoides* L., Fam. Elaeagnaceae) seed oil that revealed regenerative properties on normal skin cell. Sea buckthorn berries and other parts are known to produce fatty acids with antioxidant, anti-inflammatory and regenerative properties. The authors aim to investigate the suitability of fatty acids in the sea buckthorn seed with regenerative properties on normal skin cells. Accordingly, using preparative HPLC, they separated four fractions from the Sea Buckthorn Seed Oil followed by characterization of compounds such as linolenic acid, linoleic acid, palmitoleic acid, oleic acid and palmitic acid by LC-MS. Out of these components in four fractions, the palmitic acid enriched fraction supported cell proliferation for both keratinocytes and dermal fibroblasts. Further *in vivo* testing could suggest the suitability of this fraction in skin care cosmetic. However, this study does not support the requirement of palmitoleic acid which is useful in treating disorders related to skin hyperpigmentation as an adjuvant in formulations for the treatment of secondary infections caused by gram-positive bacteria (Weimann et al., 2018).

Zhou et al. focus on the development of instrumental method for the characterization of antibacterial compounds from *Forsythia suspensa* (Thunb.) Vahl (Fam. Oleaceae), a traditional Chinese medical herb which is well known for anti-inflammatory, antioxidant, antibacterial, anti-cancer, antivirus, anti-allergy and neuroprotective properties. In this study, the authors have utilized macroporous adsorption resin (MAR) to enrich the effective components from *F*. *suspensa* leaves where the components have been eluted with ethanol-water mixtures in increments. The authors also describe the instrumental methods such as ultra-performance liquid chromatography coupled with quadrupole time of flight mass spectrometry (UPLC/Q-TOF MS) and high-performance liquid chromatography for identification and quantification of 31 compounds including 11 glycosides, eight flavonoids, three organic acids, three phenolic, three lignans, one phenylpropanoid, one terpenoid, and one other substances. Main compounds they report in this study are glycosides and flavonoids, while the phenylethanoid glycosides have revealed the most antibacterial activity. They explore the mechanism of antibacterial compounds by damaging the bacterial cell membrane and raising bacterial cell wall permeability abnormally.

Among three review articles, Saha et al. report antimicrobial diterpenes from natural sources. As natural diterpenoids with broad spectrum of antimicrobial activity are of crucial importance, this study has reviewed the recent literatures on natural diterpenes and diterpenoids with significant antibacterial, antifungal, antiviral and antiprotozoal activities as well as their structure activity relationships. This systematic review that has focused on relevant literatures between 2017-2021 following the PRISMA guidelines summarizes 229 prospective diterpenoids with their promising antimicrobial properties including antibacterial, antiviral, antifungal and antiprotozoal properties. This systematic review will encourage researchers to look into total synthesis of lead potential natural diterpenes as well as their derivatization followed by further SAR studies for antimicrobial drug development to combat AMR.

Susanti et al. review natural products-based nanoparticles with promising antimicrobial activity. The antimicrobial action of natural products could be improved by biogenic synthesis with metal to form nanoparticles (NPs) which reveal their effectiveness in preventing or overcoming biofilm formation. Accordingly, the authors have looked into metallic nanoparticles such as silver-based nanoparticles (AgNPs), gold-based nanoparticles (AuNPs), platinum-based nanoparticles (PtNPs) and Zinc oxide-based nanoparticles (ZnONPs). Because of simplicity of overall synthesis, the researchers are encouraged to explore antimicrobial potency of metallic nanoparticles with natural products. This article reviews various types of metallic nanoparticle which have been carried out to enhance the antimicrobial potential of natural products (plant extract or constituents), overall synthetic procedure as well as the characterization of the plant-based metallic nanoparticles. Stan et al. review antimicrobial and antiviral natural compounds as well as nanocarriers for their transportation. This review has focused into antimicrobial and antiviral natural compounds from various sources- plants, microorganism and animals. Within the plants, the authors have summarized the antibacterial and antifungal activities of some plant extracts, essential oils and propolis along with the mechanism of actions. As microbes are major source of antibiotics, they have provided the antibacterial activities of some metabolites from *Streptomyces*, *Lactobacillus*, *Bacillus* and *Enterococcus*. They have also outlined antimicrobial peptides from animal sources (e.g., frogs) as well as antifungal and antiviral compounds such as flavonoids inhibiting SARS-COV-2. The importance of nanocarriers such as liposomes, drug delivery microemulsion systems, nanocapsules, solid lipid nanoparticles, polymeric micelles, dendrimers, etc. has been emphasized for drug delivery with better bioavailability, efficacy of cellular uptake/internalization, pharmacokinetic profile and reduction of toxicity of the active compounds. Some recent studies have focused on the incorporation of antimicrobial natural products into polymeric nanoparticles, niosomes and silver nanoparticles for the enhancement of their antimicrobial activity.

In conclusion, eight articles in this Research Topic provide recent research in natural products in antimicrobial drug discovery. The overall findings of both original and review articles are useful for researchers in natural products chemistry with particular interests on antimicrobial compounds from plants, microbes and animals. The problem of antimicrobial resistance and the role of natural products that have been highlighted in this topic will appeal Frontiers readers in this research area. Antimicrobial natural compounds highlighted here might be of interests of medicinal chemists for total synthesis of active compounds and making their analogues for antimicrobial drug discovery projects contributing significantly towards tackling the global issue of antimicrobial resistance. However, beyond the discoveries of new antimicrobial compounds, more inputs and efforts are also required from the Governments and healthcare authorities to educate the general public and healthcare professionals to intervene such a catastrophic issue of AMR.
